# RELIGIOSITY, DEATH EXPOSURE, AND HEALTH LITERACY AS PREDICTORS FOR ATTITUDES TOWARD MEDICAL AID IN DYING (MAID)

**DOI:** 10.1093/geroni/igad104.3025

**Published:** 2023-12-21

**Authors:** Alissa McIntyre, Amy Albright, Rebecca Allen

**Affiliations:** The University of Alabama, Tuscaloosa, Alabama, United States; The University of Alabama, Tuscaloosa, Alabama, United States; The University of Alabama, Tuscaloosa, Alabama, United States

## Abstract

Only legal in ten states (Death with Dignity, 2023), Medical aid in dying (MAID) is the current term for requesting lethal medication through a physician to end one’s life. Research has explored individuals’ characteristics who request MAID and whether they complete the prescribed medication process (Ganzini et al., 2009). However, little research explores relations between health literacy, religiosity, and individuals’ attitudes toward MAID. We hypothesized that participants with high health literacy and greater death exposure would hold greater support for MAID, while greater religiosity would be associated with lower support. Having implemented attention checks, participants (N = 265) completed measures via MTurk, assessing for health literacy, religiosity, death exposure, and attitudes toward MAID. Ages ranged from 19 to 83 (M = 37.81, SD = 12.55), with 17.7% of the sample over the age of 50. The majority were self-reported female (62.6%), non-Hispanic white (79.6%), and indicated adequate functional health literacy (82.6%). Significant correlations were observed between religiosity, attitudes toward MAID, and MAID support. Although there was no direct effect on MAID support, health literacy and death exposure remained in the path model to ensure adequate model fit. The results of this study help us understand the effect of individuals’ experiences and their attitudes toward MAID, indicating the importance of tailoring health literacy towards supporting autonomous choices in end-of-life care.

